# Efficacy and Safety of Phase 1 of Very Low Energy Ketogenic Therapy (VLEKT) in Subjects with Obesity and Mild Renal Impairment

**DOI:** 10.3390/nu17040721

**Published:** 2025-02-18

**Authors:** Ludovica Verde, Luigi Barrea, Martina Galasso, Stefania Lucà, Elisabetta Camajani, Antonio Pisani, Annamaria Colao, Massimiliano Caprio, Giovanna Muscogiuri

**Affiliations:** 1 Department of Public Health, University of Naples Federico II, Via Sergio Pansini 5, 80131 Naples, Italy; 2 Department of Medicine, Division of Endocrinology, University of Arizona, Tucson, AZ 85724, USA; 3 Dipartimento di Psicologia e Scienze Della Salute, Università Telematica Pegaso, Centro Direzionale Isola F2, Via Porzio, 80143 Naples, Italy; 4 Dipartimento di Medicina Clinica e Chirurgia, Centro Italiano per la cura e il Benessere del Paziente con Obesità (C.I.B.O), Università degli Studi di Napoli Federico II, Via Sergio Pansini 5, 80131 Naples, Italy; 5 Distretto Sanitario 67, ASL Salerno, 84085 Salerno, Italy; 6 Laboratory of Cardiovascular Endocrinology, IRCCS San Raffaele, 00166 Rome, Italy; 7 Department for the Promotion of Human Sciences and Quality of Life, San Raffaele Roma Open University, Via di Val Cannuta 247, 00166 Rome, Italy; 8 Unit of Nephrology, Federico II University of Naples, 80131 Naples, Italy; 9 Unità di Endocrinologia, Diabetologia ed Andrologia, Dipartimento di Medicina Clinica e Chirurgia, Università degli Studi di Napoli Federico II, Via Sergio Pansini 5, 80131 Naples, Italy; 10 Cattedra Unesco “Educazione Alla Salute E Allo Sviluppo Sostenibile”, Università degli Studi di Napoli Federico II, Via Sergio Pansini 5, 80131 Naples, Italy

**Keywords:** obesity, chronic kidney disease, very low energy ketogenic therapy, HOMA-IR, weight loss, anthropometric parameters, renal function

## Abstract

**Background:** Obesity impairs renal function through direct mechanisms, such as proinflammatory adipocytokine production, and indirect mechanisms, including obesity-related comorbidities. Despite the increasing prevalence of obesity and chronic kidney disease (CKD), clinical guidelines for their combined management remain lacking. Very Low Energy Ketogenic Therapy (VLEKT) has demonstrated efficacy in weight loss, but evidence on its safety and efficacy in individuals with obesity and mild renal impairment is limited. This study aimed to assess the efficacy and safety of Phase 1 of VLEKT in individuals with obesity and mild renal impairment. **Methods:** This cross-sectional study included 73 individuals with overweight or obesity (mean age 53.7 ± 8.8 years; BMI 35.3 ± 4.2 kg/m^2^) and an estimated glomerular filtration rate (eGFR) of at least 60 mL/min/1.73 m^2^ (evaluated using the CKD-EPI equation). Anthropometric (weight, BMI, and waist circumference) and biochemical parameters (fasting plasma glucose, insulin, cholesterol profile, triglycerides, AST, ALT, and urea) were collected at baseline and after 45 (±2) days of Phase 1 of VLEKT. **Results:** At baseline, 54.8% of participants had an eGFR <90 mL/min/1.73 m^2^, while 45.2% had an eGFR ≥ 90 mL/min/1.73 m^2^, with no significant differences in sex distribution. After 45 (±2) days of Phase 1 of VLEKT, both groups showed significant reductions in BMI (*p* < 0.001), waist circumference (*p* < 0.001), fasting plasma glucose (*p* ≤ 0.004), insulin (*p* < 0.001), HOMA-IR (*p* < 0.001), total cholesterol (*p* < 0.001), LDL cholesterol (*p* < 0.001), LDL/HDL ratio (*p* ≤ 0.002), triglycerides (*p* ≤ 0.009), AST (*p* ≤ 0.034), and ALT (*p* ≤ 0.009). Notably, the eGFR significantly increased in participants with an eGFR < 90 mL/min/1.73 m^2^ (*p* < 0.001), while no changes were observed in those with an eGFR ≥ 90 mL/min/1.73 m^2^. **Conclusions:** Phase 1 of VLEKT could effectively promote weight loss and metabolic improvements without compromising renal function, even in individuals with obesity and mild renal impairment. Further research is warranted to confirm the efficacy and safety of VLEKT and to assess outcomes across all protocol phases.

## 1. Introduction

Obesity represents a pressing global health problem and an acknowledged risk factor for chronic kidney disease (CKD) [1,2,3,4]. The interplay between these conditions is driven by both direct mechanisms, such as the release of proinflammatory adipocytokines, and indirect mechanisms, including obesity-related comorbidities like type 2 diabetes and hypertension [5,6,7]. These factors contribute to glomerular hyperfiltration, proteinuria, and progressive decline in glomerular filtration rate (GFR) [8,9]. Given the rising prevalence of both obesity and CKD, there is an urgent need for effective interventions that address these intertwined conditions.

Among available therapeutic strategies, the Very Low-Calorie Ketogenic Diet (VLCKD) has gained attention for its ability to induce rapid and significant weight loss in individuals with obesity [10,11]. Recently, the Italian Society of Nutraceutics (SINut) and the Italian Association of Dietetics and Clinical Nutrition (ADI) proposed a revised nomenclature for this medical nutrition therapy to avoid confusion with low-carbohydrate diets [12]. The approach, now termed Very Low Energy Ketogenic Therapy (VLEKT), is divided into distinct phases. The initial ketogenic phases (Phases 1 and 2) involve a very low-calorie diet (650–800 kcal/day), with very low carbohydrates (<30 g/day) and a minimal amount of fat, ensuring adequate protein intake to preserve lean mass. Clinical monitoring is essential during these stages. Subsequent non-ketogenic phases gradually reintroduce food groups, starting with low-glycemic-index carbohydrates, such as fruits and vegetables (Phase 3), followed by low-fat dairy (Phase 4) and, eventually, legumes and whole grains (Phase 5). Caloric intake is progressively increased across phases to support weight maintenance [12].

Despite its efficacy in weight management, VLEKT has often been considered potentially harmful for renal function due to misconceptions regarding its protein content and risks of electrolyte imbalances and increased diuresis [13]. However, recent evidence, including the Ketogenic Nutritional Therapy (KeNuT) SIE-consensus statement, emphasizes that protein intake in VLEKT should not exceed 1.5 g per kilogram of ideal body weight, making it a controlled and structured therapeutic option [14,15]. Furthermore, under appropriate medical supervision, VLEKT has been shown to have a neutral or even beneficial effect on renal function in individuals with obesity and mild renal impairment [16]. For instance, a prospective observational study by Bruci et al. demonstrated that VLEKT not only facilitated significant weight loss but also improved or preserved renal function. However, Bruci et al. followed subjects with obesity up to the end of VLEKT protocol without providing information on what occurs at the end of Phase 1 of the VLEKT protocol, which is the most critical phase for kidney function [16].

Notably, Phase 1 of VLEKT represents the hallmark that most clearly differentiates VLEKT from traditional low-calorie diets, as it induces and sustains a state of ketosis through its specific macronutrient composition and energy restriction [17]. In the study by Bruci et al., while promising results were observed, the inclusion of non-ketogenic low-calorie phases may have influenced or even masked the renal effects specifically attributable to the ketogenic phase [16]. This highlights the need to isolate and evaluate the effects of Phase 1 of VLEKT (i.e., the ketogenic phase) to better understand its impact on renal function and metabolic outcomes.

Considering this background, the aim of this observational cross-sectional study was to evaluate the efficacy and safety of Phase 1 of VLEKT in individuals with overweight or obesity and mild renal impairment.

## 2. Materials and Methods

### 2.1. Design and Setting

This observational cross-sectional study was conducted between May 2022 and March 2024 at the Unit of Endocrinology, Obesity Unit (Centro Italiano per la Cura e il Benessere del Paziente con Obesità, “C.I.B.O.” and European Association for the Study of Obesity, Collaborating Centre for Obesity Management, “EASO-COMs”), Clinical Medicine and Surgery Department, University of Naples Federico II, Naples, Italy. Participants were identified through electronic health records from our outpatient clinic database. The study protocol was approved by the Federico II Ethics Committee (protocol number 50/20). All participants provided informed consent after being informed of the study’s objectives and design.

### 2.2. Study Population

**Figure 1:**
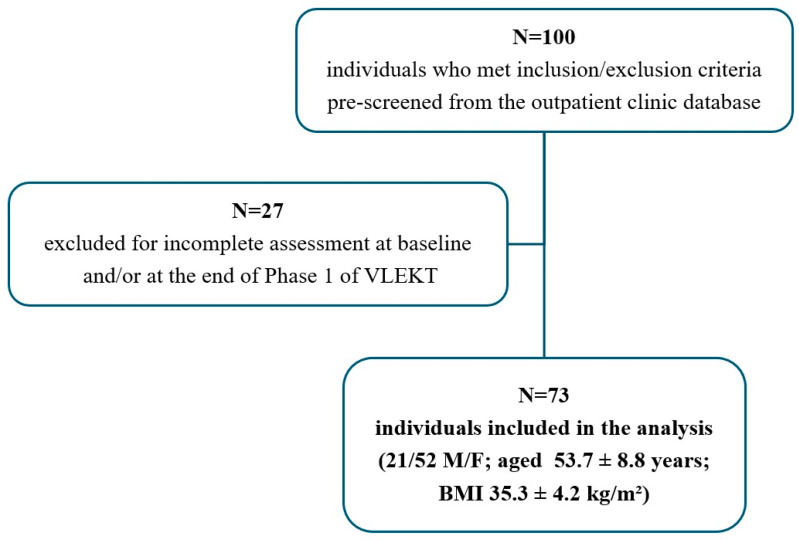
The flowchart of individuals screened and included in the analysis. VLEKT, very low energy ketogenic therapy; BMI, body mass index.

A total of 73 individuals with overweight or obesity, aged between 20 and 69 years, were screened from the outpatient clinic database. The flowchart of individuals screened and included in the analysis is represented in Figure 1. The inclusion criteria were a BMI ≥ 25.0 kg/m^2^, an estimated GFR (eGFR) ≥ 60 mL/min/1.73 m^2^, and completion of at least 45 (±2) days of Phase 1 of VLEKT. Exclusion criteria included severe renal impairment (eGFR <60 mL/min/1.73 m^2^), use of glucose-lowering medications, use of medications affecting renal function (such as SGLT-2 inhibitors or high-dose RAAS inhibitors), uncontrolled hypertension, active liver disease, pregnancy or lactation, recent significant weight loss or participation in another dietary intervention, malabsorption disorders (e.g., celiac disease or inflammatory bowel disease), history of eating disorders, severe cardiovascular disease (e.g., recent myocardial infarction or heart failure), and incomplete medical records preventing adequate data analysis.

### 2.3. Study Protocol

At baseline, all individuals underwent a comprehensive medical and nutritional evaluation. An endocrinologist conducted a detailed clinical history review, excluding contraindications to VLEKT according to the European Association for the Study of Obesity (EASO) [17]. Dyslipidemia and type 2 diabetes were assessed at baseline using a questionnaire with binary response options (YES/NO). Anthropometric data, including weight, height, and waist circumference, were measured, and blood samples were obtained. Individuals also received individualized nutritional counseling from a qualified nutritionist, who tailored the VLEKT protocol to each participant. Follow-up assessments occurred at the end of Phase 1 of VLEKT. During this visit, individuals underwent repeat anthropometric and laboratory evaluations.

### 2.4. Anthropometric Measurements

Anthropometric measurements were conducted at both baseline and at the end of Phase 1 of VLEKT by the same nutritionist to maintain consistency. All assessments took place early in the morning after an overnight fast, with individuals dressed in light clothing and barefoot. Weight was measured using a calibrated beam scale (Seca 711; Seca, Hamburg, Germany) with an accuracy of 0.1 kg, while height was determined using a wall-mounted stadiometer with a precision of 0.5 cm. BMI was calculated as weight (kg) divided by height squared (m^2^), and individuals were categorized into normal weight, overweight, or obesity grades I, II, or III in accordance with the WHO. Waist circumference was assessed using a non-stretchable measuring tape, accurate to 0.1 cm, at the midpoint between the lowest rib and the iliac crest, or at the umbilicus if the narrowest point was not accessible.

### 2.5. Laboratory Assessments

Laboratory evaluations were performed at baseline and at the end of Phase 1 of VLEKT. Blood samples were obtained through venipuncture following an overnight fast, between 8:00 and 10:00 a.m., and processed by standard protocols. Measurements included fasting plasma glucose and insulin, total cholesterol, LDL and HDL cholesterol, triglycerides, AST, ALT, and urea. Additionally, the LDL/HDL ratio was calculated.

### 2.6. Renal Function Assessment

Renal function was evaluated using the Chronic Kidney Disease Epidemiology Collaboration (CKD-EPI) equation [18] to estimate eGFR. Following KDIGO 2024 guidelines [19], CKD was categorized into five stages (G1–G5) based on eGFR levels. Only participants with normal (G1) or mildly reduced (G2) eGFRs were included, as per EASO guidelines [17].

### 2.7. Insulin Resistance Assessment

Insulin resistance was evaluated using the Homeostatic Model Assessment of Insulin Resistance (HOMA-IR), derived by dividing the product of fasting plasma glucose (mmol/L) and insulin (mU/L) by 22.5 [20].

### 2.8. Nutritional Intervention

All individuals included in the analysis underwent VLEKT protocol using a commercial meal replacement program (New Penta Srl, Cuneo, Italy). Phase 1 of VLEKT was characterized by an energy intake of <800 kcal/day, with a macronutrient composition comprising 13% carbohydrates (<30 g/day), ~43% proteins (1.3 g/kg ideal body weight), and ~44% fats. High-biological-value protein sources (whey, soy, eggs, and peas) were used for meal replacements [17]. Given the restrictive nature of the diet, individuals received specifically formulated supplements designed to meet the recommended daily allowances (RDA) for key micronutrients. The supplementation provided the following daily intake: magnesium (240 mg), potassium (3.9 g), calcium (1000 mg), vitamin C (105 mg), vitamin E (13 mg), selenium (55 μg), vitamin A (700 μg), vitamin B5 (30 μg), vitamin B6 (1.3 mg), vitamin B1 (1.2 mg), vitamin B2 (1.6 mg), folic acid (400 μg), vitamin B12 (2.4 μg), vitamin D (15 μg), and omega-3 (250 mg of EPA-DHA). These supplements were administered in two daily doses to ensure optimal absorption and adherence [17].

### 2.9. Adherence to the Nutritional Intervention

Adherence was monitored as YES/NO through weekly telephone consultation, during which individuals provided β-hydroxybutyrate levels measured in capillary blood using test strips (Optium Xceed Blood Glucose and Ketone Monitoring System; Abbott Laboratories, Chicago, IL, USA). Individuals were instructed to perform these measurements in the morning, under fasting conditions, and at consistent times.

### 2.10. Power Analysis

The power of the sample was calculated by the difference of means ± standard deviation (SD) of the eGFR score pre- and at the end of Phase 1 of VLEKT (90.23 ± 14.57 vs. 92.58 ± 13.69; respectively). The minimum number of cases required was 70 individuals. The calculated power size was 95%, with a type I (α) error of 0.05 (95%), and a type II (β) error of 0.05. The calculations of sample size and power were performed while using a sample size calculator, Clinical Calc (https://clincalc.com/stats/samplesize.aspx (accessed on 10 April 2022)).

### 2.11. Statistical Analysis

Only individuals who completed both visits (baseline and after 45 (±2) days of Phase 1 of VLEKT) and assessments were included in the analysis. MedCalc^®^ software version 22.021 and SPSS version 22.0 were used to process the data. Categorical variables were reported as frequencies (n, %), and continuous variables were shown as mean ± standard deviation (SD). The Kolmogorov–Smirnov test was used to determine whether the data distribution was normal. Logarithmic transformation was applied to skewed variables. Paired Student’s *t*-tests were used for continuous variables to compare baseline and post-intervention values, while the chi-square (χ^2^) test was employed for categorical variables. A *p*-value < 0.05 was considered statistically significant.

## 3. Results

A total of 73 individuals with overweight or obesity, who met the inclusion and exclusion criteria, were included in the study analysis. The mean age was 53.7 ± 8.8 years, with 71.2% (*n* = 52) of females and 28.8% (*n* = 21) of males, and a mean BMI of 35.3 ± 4.2 kg/m^2^. Of these individuals, 9.6% were smokers, 16.4% had type 2 diabetes, and 45.2% had dyslipidemia.

Table 1 presents the anthropometric and biochemical parameters before and after 45 days (±2 days) of Phase 1 of VLEKT in the study population.

As expected, following Phase 1 of VLEKT, significant reductions were observed in anthropometric parameters. BMI decreased from 35.3 ± 4.2 kg/m^2^ at baseline to 32.2 ± 4.2 kg/m^2^ after 45 days (±2 days) (*p* < 0.001), and waist circumference reduced from 105.5 ± 10.0 cm to 98.5 ± 10.1 cm (*p* < 0.001). Biochemical parameters also showed notable decreases. Fasting plasma glucose decreased from 102.1 ± 15.7 mg/dL to 91.9 ± 12.4 mg/dL (*p* < 0.001), insulin from 18.0 ± 10.2 µIU/mL to 8.3 ± 3.8 µIU/mL (*p* < 0.001), and HOMA-IR from 4.7 ± 3.3 to 1.9 ± 1.0 (*p* < 0.001). Total cholesterol dropped from 214 ± 39 mg/dL to 174 ± 35 mg/dL (*p* < 0.001), LDL cholesterol from 135 ± 33 mg/dL to 104 ± 28 mg/dL (*p* < 0.001), and the LDL/HDL ratio from 2.7 ± 1.1 to 2.1 ± 0.7 (*p* < 0.001). Triglycerides decreased from 132 ± 73 mg/dL to 93 ± 33 mg/dL (*p* < 0.001), AST from 24 ± 11 U/L to 20 ± 9 U/L (*p* = 0.001), and ALT from 30 ± 16 U/L to 24 ± 14 U/L (*p* < 0.001), all showing significant reductions.

Table 2 presents anthropometric and biochemical parameters before and after 45 days (±2 days) of Phase 1 of VLEKT according to the eGFR. Individuals were divided into two groups based on eGFR values of <90 mL/min/1.73 m^2^ or ≥90 mL/min/1.73 m^2^. Specifically, 40 individuals (54.8%) had an eGFR < 90 mL/min/1.73 m^2^, while 33 individuals (45.2%) had an eGFR ≥ 90 mL/min/1.73 m^2^. No significant differences in sex distribution were observed between groups (*p* = 0.438).

In individuals with an eGFR < 90 mL/min/1.73 m^2^, significant reductions in anthropometric parameters were observed from baseline to the end of Phase 1 of VLEKT. Specifically, BMI decreased from 34.2 ± 3.9 to 31.0 ± 3.8 kg/m^2^ (*p* < 0.001), and waist circumference decreased from 104.1 ± 10.2 to 97.1 ± 10.6 cm (*p* < 0.001). Additionally, fasting plasma glucose (102.7 ± 16.5 vs. 90.4 ± 10.3 mg/dL, *p* < 0.001), insulin (17.0 ± 9.4 vs. 8.4 ± 3.3 µIU/mL, *p* < 0.001), HOMA-IR (4.5 ± 3.5 vs. 1.9 ± 0.9, *p* < 0.001), total cholesterol (215 ± 34 vs. 173 ± 33 mg/dL, *p* < 0.001), LDL cholesterol (134 ± 29 vs. 102 ± 26 mg/dL, *p* < 0.001), LDL/HDL ratio (2.7 ± 1.0 vs. 2.0 ± 0.7, *p* < 0.001), and triglycerides (122 ± 35 vs. 88 ± 25 mg/dL, *p* < 0.001) all exhibited significant decreases. Moreover, participants with an eGFR < 90 mL/min/1.73 m^2^ showed significant decreases in AST (24 ± 10 vs. 21 ± 9 U/L, *p* = 0.034) and ALT (27 ± 11 vs. 23 ± 9 U/L, *p* = 0.009) levels.

In individuals with an eGFR ≥ 90 mL/min/1.73 m^2^, significant reductions in anthropometric parameters were also observed. BMI decreased from 36.6 ± 4.3 to 33.5 ± 4.3 kg/m^2^ (*p* < 0.001), and waist circumference decreased from 107.1 ± 9.7 to 100.3 ± 9.4 cm (*p* < 0.001). Significant reductions were noted in fasting plasma glucose (101.3 ± 15.0 vs. 93.7 ± 14.5 mg/dL, *p* = 0.004), fasting plasma insulin (19.1 ± 11.2 vs. 8.1 ± 4.4 µIU/mL, *p* < 0.001), HOMA-IR (4.9 ± 3.0 vs. 1.9 ± 1.1, *p* < 0.001), total cholesterol (213 ± 45 vs. 175 ± 37 mg/dL, *p* < 0.001), LDL cholesterol (137 ± 38 vs. 107 ± 30 mg/dL, *p* < 0.001), LDL/HDL ratio (2.8 ± 1.2 vs. 2.2 ± 0.8, *p* = 0.002), and triglycerides (144 ± 101 vs. 99 ± 30 mg/dL, *p* = 0.009). Additionally, participants with an eGFR ≥ 90 mL/min/1.73 m^2^ demonstrated significant decreases in AST (24 ± 11 vs. 20 ± 11 U/L, *p* = 0.016) and ALT (32 ± 20 vs. 25 ± 18 U/L, *p* = 0.004) levels.

Notably, a significant increase in eGFR was observed in the group with eGFRs < 90 mL/min/1.73 m^2^ (79.1 ± 8.3 vs. 85.6 ± 12.0, *p* < 0.001), while no significant changes were observed in the group with eGFRs ≥ 90 mL/min/1.73 m^2^.

## 4. Discussion

This study assessed the efficacy and safety of Phase 1 of VLEKT in individuals with overweight or obesity and mild renal impairment. The findings revealed significant improvements in anthropometric and metabolic parameters, providing evidence that VLEKT is both an effective weight loss strategy and a potentially safe option for managing obesity-related complications, even in individuals with early-stage kidney disease. Individuals demonstrated significant reductions in BMI, waist circumference, fasting plasma glucose, insulin, HOMA-IR, total cholesterol, LDL cholesterol, LDL/HDL, triglycerides, AST, and ALT levels after 45 days (±2 days) of Phase 1 of VLEKT. These results align with previous studies highlighting the rapid metabolic improvements achievable with VLEKT in individuals with obesity [21,22]. Notably, the observed drop in HOMA-IR highlights how well VLEKT works to decrease insulin resistance, which is a critical component in lowering the risk of cardiovascular disease and type 2 diabetes.

In our study, a significant increase in eGFR was observed in the group with eGFRs < 90 mL/min/1.73 m^2^, while no significant changes were observed in the group with eGFRs ≥ 90 mL/min/1.73 m^2^, confirming that Phase 1 of VLEKT did not adversely affect renal function. These findings were particularly important given the common concerns regarding ketogenic diets and kidney health [4,5,13].

Similarly, Bruci et al. observed an improvement in eGFR in a study population of 92 individuals with obesity (mean BMI: 33.8 ± 5.8 kg/m^2^), including those with mild chronic kidney disease (MCKD; eGFR: 60–89 mL/min/1.73 m^2^) and normal kidney function (NKF; eGFR ≥ 90 mL/min/1.73 m^2^) [16]. Their study evaluated participants over the full duration of the VLEKT protocol, encompassing the active, re-education, and maintenance phases [16]. In contrast, our study focused exclusively on Phase 1 of VLEKT, which is characterized by ketosis—a state driven by the production and utilization of ketone bodies, particularly β-hydroxybutyrate [23].

This distinction in study design underscores the rationale behind our approach: by isolating the ketogenic phase, we aimed to capture the specific effects of ketosis on renal function and metabolic parameters. Emerging evidence suggests that ketone bodies, particularly β-hydroxybutyrate, play a protective role in renal physiology [24,25]. In kidney cells, β-hydroxybutyrate has been demonstrated to improve energy metabolism and mitochondrial efficiency while lowering oxidative stress, inflammation, and fibrosis [24]. These protective mechanisms provide a theoretical basis to explain why Phase 1 of VLEKT, despite inducing ketosis, did not negatively impact renal function in our study population.

By focusing on Phase 1 of VLEKT, our study highlights the renal safety of the ketogenic state itself, which has often been carefully addressed due to misconceptions about its impact on kidney health [26]. The lack of adverse effects observed in our study provides valuable insights into the safety of VLEKT during this critical phase, while also paving the way for future research to evaluate the long-term renal outcomes across all phases of the protocol.

While our findings are promising, this study has limitations. The observational design does not provide information on pathophysiological mechanisms underlying any link between VLEKT and kidney function. Additionally, renal function was estimated using the CKD-EPI equation rather than direct measurement methods such as 24 h urinary collection, as it may not provide a completely accurate representation of renal function in subjects with mild renal impairment (due to the creatinine-blind range) [27]. Given the observational nature of our study, future randomized controlled trials should evaluate the effects of each phase of VLEKT on renal function to provide a more comprehensive understanding of its long-term safety and efficacy.

## 5. Conclusions

In conclusion, Phase 1 of VLEKT appears to be a safe and effective nutritional intervention for managing obesity and its metabolic complications, even in individuals with early-stage renal impairment. Future research should confirm its efficacy and safety and explore the long-term effects of all phases of VLEKT to better define its role in managing obesity-related kidney complications.

## Figures and Tables

**Table 1:** Anthropometric and biochemical parameters before and after 45 days (±2 days) of Phase 1 of VLEKT in the study population.

Parameters	Baseline (*n* = 73)	After 45 Days (±2 Days) of Phase 1 (*n* = 73)	*p*-Value	Δ%
Anthropometrical parameters				
Weight (kg)	97.7 ± 13.3	89.0 ± 12.5	**<0.001**	−8.9 ± 2.9
BMI (kg/m^2^)	35.3 ± 4.2	32.2 ± 4.2	**<0.001**	−8.9 ± 3.0
Normal weight (*n*,%)	0	1 (1.4)	χ^2^= 0.01, *p* = 1.000	
Overweight (*n*,%)	6 (8.2)	23 (31.5)	χ^2^= 11.02, ***p* < 0.001**	
Obesity I class (*n*,%)	30 (41.1)	30 (41.1)	χ^2^= 0.03, *p* = 0.866	
Obesity II class (*n*,%)	26 (35.6)	16 (21.9)	χ^2^= 2.71, *p* = 0.099	
Obesity III class (*n*,%)	11 (15.1)	3 (4.1)	χ^2^= 3.87, ***p* = 0.048**	
WC (cm)	105.5 ± 10.0	98.5 ±10.1	**<0.001**	−6.6 ± 3.1
Biochemical parameters				
Fasting plasma glucose (mg/dL)	102.1 ± 15.7	91.9 ± 12.4	**<0.001**	−8.9 ± 12.1
Insulin (µIU/mL)	18.0 ± 10.2	8.3 ± 3.8	**<0.001**	−47.3 ± 26.7
HOMA-IR	4.7 ± 3.3	1.9 ± 1.0	**<0.001**	−50.7 ± 29.2
Total cholesterol (mg/dL)	214 ± 39	174 ± 35	**<0.001**	−19.3 ± 15.2
HDL cholesterol (mg/dL)	53 ± 16	52 ± 14	0.253	−0.0 ± 21.2
LDL cholesterol (mg/dL)	135 ± 33	104 ± 28	**<0.001**	−22.2 ± 15.7
LDL/HDL	2.7 ± 1.1	2.1 ± 0.7	**<0.001**	−18.7 ± 23.7
Triglycerides (mg/dL)	132 ± 73	93 ± 33	**<0.001**	−22.9 ± 25.7
AST (U/L)	24 ± 11	20 ± 9	**0.001**	−10.1 ± 27.3
ALT (U/L)	30 ± 16	24 ± 14	**<0.001**	−12.1 ± 28.7
eGFR (mL/min/1.73 m^2^)	90.2 ± 14.6	92.5 ± 13.7	0.062	3.6 ± 12.7
<90 mL/min/1.73 m^2^ (*n*,%)	40 (54.8)	29 (39.7)	χ^2^ = 2.75, *p* = 0.097	
≥90 mL/min/1.73 m^2^ (*n*,%)	33 (45.2)	44 (60.3)		
Urea (mg/dL)	37 ± 10	37 ± 12	0.987	1.3 ± 27.3

BMI, body mass index; WC, waist circumference; HOMA-IR, homeostatic model of assessment of insulin resistance; HDL, high density lipoprotein; LDL, low density lipoprotein; AST, aspartate aminotransferase; ALT, alanine aminotransferase; eGFR, estimated glomerular filtration rate. A *p* value in bold type denotes a significant difference (*p* < 0.05).

**Table 2:** Anthropometric and biochemical parameters before and after 45 days (±2 days) of Phase 1 of VLEKT according to eGFR.

Parameters	<90 mL/min/1.73 m^2^			≥90 mL/min/1.73 m^2^		
	Baseline (*n* = 40)	After 45 Days (±2 Days) of Phase 1 (*n* = 40)	*p*-Value	Baseline (*n* = 33)	After 45 Days (±2 Days) of Phase 1 (*n* = 33)	*p*-Value
Anthropometrical parameters						
Weight (kg)	95.9 ± 12.0	86.9 ± 10.9	**<0.001**	99.9 ± 14.6	91.4 ± 14.0	**<0.001**
BMI (kg/m^2^)	34.2 ± 3.9	31.0 ± 3.8	**<0.001**	36.6 ± 4.3	33.5 ± 4.4	**<0.001**
Normal weight (*n*,%)	0	1 (2.5%)	**0**	0	0	
Overweight (*n*,%)	5 (12.5%)	15 (37.5%)	χ^2^ = 5.40, ***p* = 0.020**	1 (3.0%)	8 (24.2%)	χ^2^ = 4.63, ***p* = 0.031**
Obesity I class (*n*,%)	18 (45.5%)	20 (50.0%)	χ^2^ = 0.05, *p* = 0.823	12 (36.4%)	10 (30.3%)	χ^2^ = 0.07, *p* = 0.794
Obesity II class (*n*,%)	14 (35.0%)	3 (7.5%)	χ^2^ = 7.47, ***p* = 0.006**	12 (36.4%)	13 (39.4%)	χ^2^ = 0.00, *p* = 1.000
Obesity III class (*n*,%)	3 (7.5%)	1 (2.5%)	χ^2^ = 0.26, *p* = 0.608	8 (24.2%)	2 (6.1%)	χ^2^ = 2.95, *p* = 0.086
WC (cm)	104.1 ± 10.2	97.1 ± 10.6	**<0.001**	107.1 ± 9.7	100.3 ± 9.4	**<0.001**
Biochemical parameters						
Fasting plasma glucose (mg/dL)	102.7 ± 16.5	90.4 ± 10.3	**<0.001**	101.3 ± 15.0	93.7 ± 14.5	**0.004**
Insulin (µIU/mL)	17.0 ± 9.4	8.4 ± 3.3	**<0.001**	19.1 ± 11.2	8.1 ± 4.4	**<0.001**
HOMA-IR	4.5 ± 3.5	1.9 ± 0.9	**<0.001**	4.9 ± 3.0	1.9 ± 1.1	**<0.001**
Total cholesterol (mg/dL)	214 ± 34	173 ± 33	**<0.001**	213 ± 45	175 ± 37	**<0.001**
HDL cholesterol (mg/dL)	54 ± 18	54 ± 15	0.503	51 ± 14	50 ± 13	0.316
LDL cholesterol (mg/dL)	133 ± 28	102 ± 26	**<0.001**	137 ± 38	107 ± 30	**<0.001**
LDL/HDL	2.7 ± 1.0	2.0 ± 0.7	**<0.001**	2.8 ± 1.2	2.2 ± 0.8	**0.002**
Triglycerides (mg/dL)	122 ± 35	88 ± 25	**<0.001**	144 ± 101	99 ± 30	**0.009**
AST (U/L)	24 ± 10	21 ± 9	**0.034**	24 ± 11	20 ± 11	**0.016**
ALT (U/L)	27 ± 11	23 ± 9	**0.009**	32 ± 20	25 ± 18	**0.004**
eGFR (mL/min/1.73 m^2^)	79.1 ± 8.3	85.6 ± 12.0	**<0.001**	103.8 ± 7.0	101.0 ± 10.5	0.072
Urea (mg/dL)	40 ±10	38 ± 11	0.393	34 ± 8	36 ± 13	0.523

BMI, body mass index; WC, waist circumference; HOMA-IR, homeostatic model of assessment of insulin resistance; HDL, high density lipoprotein; LDL, low density lipoprotein; AST, aspartate aminotransferase; ALT, alanine aminotransferase; eGFR, estimated glomerular filtration rate. A *p* value in bold type denotes a significant difference (*p* < 0.05).

## Data Availability

The data presented in this study are available on request from the corresponding author due to ethical reason.
